# Preclinical evaluation of an ^111^In/^225^Ac theranostic targeting transformed MUC1 for triple negative breast cancer

**DOI:** 10.7150/thno.38236

**Published:** 2020-05-25

**Authors:** Vanessa J Kelly, Shu-Ta Wu, Vijay Gottumukkala, Richard Coelho, Keryn Palmer, Surabhi Nair, Timothy Erick, Rahul Puri, Ohad Ilovich, Pinku Mukherjee

**Affiliations:** 1 Invicro, LLC, Boston, MA, USA.; 2 Department of Biological Sciences, University of North Carolina, Charlotte, NC, USA.; 3 OncoTAb, Inc., Charlotte, NC, USA.

**Keywords:** MUC1, Targeted Alpha Radiotherapy, Radioimmunotherapy, Theranostic

## Abstract

**Rationale**: Transformed MUC1 (tMUC1) is a cancer-associated antigen that is overexpressed in >90% of triple-negative breast cancers (TNBC), a highly metastatic and aggressive subtype of breast cancer. TAB004, a murine antibody targeting tMUC1, has shown efficacy for the targeted delivery of therapeutics to cancer cells. Our aim was to evaluate humanized TAB004 (hTAB004) as a potential theranostic for TNBC.

**Methods**: The internalization of hTAB004 in tMUC1 expressing HCC70 cells was assessed via fluorescent microscopy. hTAB004 was DOTA-conjugated and radiolabeled with Indium-111 or Actinium-225 and tested for stability and tMUC1 binding (ELISA, flow cytometry). Lastly, *in vivo* biodistribution (SPECT-CT), dosimetry, and efficacy of hTAB004 were evaluated using a TNBC orthotopic mouse model.

**Results**: hTAB004 was shown to bind and internalize into tMUC1-expressing cells. A production method of ^225^Ac-DOTA-hTAB004 (yield>97%, RCP>97% SA=5 kBq/µg) and ^111^In-DOTA-hTAB004 (yield>70%, RCP>99%, SA=884 kBq/µg) was developed. The labeled molecules retained their affinity to tMUC1 and were stable in formulation and mouse serum. In NSG female mice bearing orthotopic HCC70 xenografts, the *in vivo* tumor concentration of ^111^In-DOTA-hTAB004 was 65 ± 15 %ID/g (120 h post injection). A single ^225^Ac-DOTA-hTAB004 dose (18.5 kBq) caused a significant reduction in tumor volume (P<0.001, day 22) and increased survival compared to controls (P<0.007). The human dosimetry results were comparable to other clinically used agents.

**Conclusion**: The results obtained with hTAB004 suggest that the ^111^In/^225^Ac-DOTA-hTAB004 combination has significant potential as a theranostic strategy in TNBC and merits further development toward clinical translation.

## Introduction

Triple-negative breast cancers (TNBC) represent approximately 15% of all breast cancer cases 1, and are associated with poor prognosis, a greater risk of relapse, and high mortality rates following relapse 2. TNBC is defined by a lack of expression of estrogen, progesterone, and HER2/neu receptors which means TNBC patients have not benefited from the recent wave of receptor targeted therapies (Trastuzumab, Pertuzumab) 3,4. Consequently, new treatment options for TNBC are needed.

A promising therapeutic target for TNBC is the transformed Mucin 1 (tMUC1) which is overexpressed in >90% of TNBCs 5,6. Normal Mucin 1 (MUC1) is a transmembrane mucin glycoprotein that is expressed on all glandular epithelial cells. In normal epithelial cells, MUC1 is expressed on the apical surface, and is heavily glycosylated such that the core protein is sequestered by the carbohydrates. In tumor cells, including breast tumors, MUC1 is transformed (tMUC1), hypoglycosylated, overexpressed and its distribution is altered 7. Within the tMUC1 tandem repeat sequence there is an altered glycosylated epitope that is bound with high affinity (20 pM) by a murine antibody termed TAB004 8. The epitope is accessible for antigenic detection in tMUC1 but is masked in normal MUC1 by large branches of glycosylation 9-11. Once TAB004 binds to tMUC1 it has been shown to internalize 12. Together, the expression profile of tMUC1 in TNBC, the high binding selectivity of TAB004 to a tMUC1 epitope and post-binding internalization make TAB004 an excellent therapeutic candidate for TNBC and, in particular, for use as a theranostic agent.

The basic concept of theranostic agents is to form a targeting molecule-radioisotope complex using a high affinity binder that targets a tumor-specific antigen (i.e. TAB004 and tMUC1). The radioisotope in the complex may be used for both diagnostic and therapy, or alternatively, a combination of interchangeable radioisotopes is needed (one for imaging, one for therapy). Once the theranostic agent binds to its target antigen, the complex may remain on the cell surface or is internalized. Internalization is an important component of theranostics that utilize alpha emitting isotopes for therapy. Specifically, the complex decay series of most long-lived alpha emitters, their high recoil energy and the short path length of alpha particles mean that intracellular decay is preferable 13. Alpha emitters are delivered in low doses (<1 kBq/kg) and have low yields of imageable gamma emissions making their direct imaging challenging. Subsequently, if utilizing alpha emitters for the therapy a gamma/positron emitting companion diagnostic is required to assess the biodistribution and dosimetry prior to therapy initiation.

The objective of this work was to evaluate the potential of TAB004 as a translatable theranostic radiopharmaceutical for TNBC. To this end a humanized version of TAB004 (hTAB004) was used to form an antibody-radioisotope complex by conjugating hTAB004 with a chelator (DOTA) to allow for radiolabeling with either Indium-111 (for SPECT imaging) or Actinium-225 (for alpha radiotherapy). The resultant compounds were evaluated for stability in mouse serum and ^111^In-DOTA-hTAB004's biodistribution was evaluated with *in vivo* SPECT imaging studies in HCC70 orthotopic tumor bearing mice. The biodistribution results were used to calculate dosimetry estimates for the use of ^111^In-DOTA-hTAB004 in humans. We also used these results to calculate the mouse dosimetry estimates of ^225^Ac-DOTA-hTAB004 in the same model. These dosimetry estimates allowed us to deliver a safe dose of ^225^Ac-DOTA-hTAB004 to orthotopic HCC70 tumor bearing mice that resulted in both significant tumor shrinkage and significantly greater survival. Taken together, our pre-clinical studies support the utility of hTAB004 as a potential theranostic for TNBC.

## Methods

### Antibody Humanization

Humanization of murine TAB004 was performed via combining hybrid sequences that fuse select parts of the parental antibody sequence with the human framework sequences of the light and heavy chains with the help of 3D modelling. This process produced 9 candidate antibodies that were tested for expression level and binding affinity using flow cytometry and ELISA (data not shown). The clone with the most similar binding affinity to the murine TAB004 was selected.

### Radiolabeling and Stability

All chemicals were purchased from Sigma-Aldrich (St Louis, MO, USA) and used without further purification. DOTA-NHS-ester was purchased from Macrocyclics (Plano, TX, USA). Buffers were made with metal free water (18 mΩ•cm).

hTAB004 (2.38 mg, 1 mL, 150 kDa) was incubated with EDTA (50 mM) for 15 min at room temperature and buffer exchanged into HEPES buffer (0.1 M, pH 8.5) using a 50 kDa Amicon ultra centrifugal filter. A freshly made solution of DOTA-NHS-ester in DMSO (51 µg, 5 mg/mL) was added to the protein to afford a 20:1 DOTA:hTAB004 molar ratio. The reaction was mixed at 37 °C for 30 min, followed by overnight incubation at 4 °C. The crude mixture was buffer exchanged into ammonium acetate (0.1 M, pH 5.58) to give DOTA-hTAB004. Final protein content of the DOTA-hTAB004 and reaction yield (800 µg, 34% yield) was calculated using a NanoDrop light spectrophotometer (Thermo Fisher Scientific Inc, Waltham, MA). The DOTA-to-hTAB004 ratio was determined via liquid chromatography-mass spectrometry (LC-MS). See Online Data Supplement for detailed Methods (Table S1).

The method for radiolabeling DOTA-hTAB004 with either Indium-111 (Cardinal Health, Dublin, OH, USA) or Actinium-225 (ORNL, Oak Ridge, TN, USA) was developed such that both isotopes would label under the same conditions. Specifically, the radiometal (Indium-111 or Actinium-225) was diluted in MES buffer (0.5 M, pH 5.53) and DOTA-hTAB004 was added to the reaction mixture. The reaction was incubated at 37 °C for up to 2 h and monitored via iTLC (Biodex TLC strip, 0.1 M acetate buffer with 25 mM EDTA). ^225^Ac-DOTA-hTAB004 was obtained at >95% radiochemical purity and was diluted with 5% ascorbic acid in PBS prior to use. The crude ^111^In-DOTA-hTAB004 product had a radiochemical purity <95% and so was buffer exchanged into PBS using an Amicon ultra centrifugal filter to remove any unchelated metal prior to use (>99% radiochemical purity). Both compounds were diluted with hTAB004 to achieve the required specific activity for *in vivo* work. Similar labeling conditions were applied with Indium-115 and Lanthanum-139 to produce the non-radioactive derivatives of the radiopharmaceuticals (^115^In-DOTA-hTAB004 and ^139^La-DOTA-hTAB004) in support of the flow cytometry and ELISA studies.

The stability of ^111^In-DOTA-hTAB004 and ^225^Ac-DOTA-hTAB004 was assessed by incubating the material in either formulation at 2-7 °C or with mouse serum (1:3, 37 °C). Samples of each mixture were removed and analyzed via size exclusion chromatography over a period of 120 h. For samples of ^225^Ac-DOTA-hTAB004 60 s (1 mL) fractions were collected off the column and each fraction gamma counted 24 h after collection. Plots of CPM vs. retention time were generated and area under the curve was computed for all visible peaks via trapezoidal integration.

### Antigen Binding and Internalization

Sandwich ELISA was used to determine if the binding of the hTAB004 derivatives (DOTA-hTAB004, ^115^In-DOTA-hTAB004, ^139^La-DOTA-hTAB004) was equivalent to the parent hTAB004. The assay allowed for the quantification of the optical density (OD) value relative to the concentration of the hTAB004 derivative. A detailed description of the sandwich ELISA is included in the Online Data Supplement.

Flow cytometry was used to confirm that the hTAB004 derivatives bind to tMUC1 expressing cells (HCC70 cells): Samples of 1 × 10^5^ HCC70 cells were suspended in 100 µL flow buffer (1×PBS with 1% FBS) and treated with 0.2 μg and 1 μg of each primary antibody/clone. Cells were then incubated on ice in the dark for 30 min, washed with flow buffer and then incubated with 1:100 dilution of FITC-anti-human IgG secondary antibody (Invitrogen, Waltham, MA) on ice in the dark for 30 min. Lastly, the cells were washed with flow buffer, and analyzed via flow cytometry (BD Fortessa, Franklin Lakes, NJ, USA). All data was analyzed using the FlowJo program (Ashland, OR, USA).

Confocal Microscopy was performed to determine if hTAB004 internalizes into HCC70 cells. Specifically, HCC70 cells were plated in 4-well glass slides (MilliporeSigma, Burlington, MA, USA) and treated with 5 µg of hTAB004 conjugated to HiLyte Fluor 647 (HL647-hTAB004) at 2-7 °C and 37 °C for 0, 5, 10, 20, 30, 40, 50, and 60 min. Cells were also treated with 5 μg/mL Wheat Germ Agglutinin-Alexa Fluor 488 conjugate (ThermoFisher Scientific, Waltham, MA, USA) for 10 min, washed with PBS and fixed with formaldehyde (4%) for 30 min at room temperature. Prolong Diamond Antifade Mountant with DAPI (ThermoFisher Scientific, Waltham, MA, USA) was applied to mount coverslips and images acquired on a confocal microscope (Olympus Fluoview FV 1000).

### Animal model

Animal studies were performed at the University of North Carolina (UNC, Charlotte NC, USA) and Invicro LLC (Boston, MA, USA) with the approval of the UNC Department of Biological Sciences and the Invicro Institutional Animal Care and Use Committee, respectively. Mice were group housed with enrichment. Housing was kept between 18-26 °C, 50 ± 20% humidity and intermittent light and dark cycles of 12 h with food and water available *ad libitum*.

Two strains of mice were used for *in vivo* studies. Specifically, biodistribution studies were performed in NOD scid gamma (NSG) mice and efficacy studies in athymic nude mice. NSG mice have higher sensitivities to radiation 14 and therefore were not used for the efficacy studies. Animals were inoculated, orthotopically, with HCC70 tumors (for details see Online Data Supplement) and monitored for tumor growth via caliper measurements. At 27 days post inoculation mice with tumor volumes in the target range (~150-300 mm^2^) were randomized (N=3 NSG, N=10 athymic nude) into their respective groups and the *in vivo* studies initiated.

### SPECT/CT

HCC70 orthotopic tumor bearing NSG mice (n=3) were injected intravenously with ^111^In-DOTA-hTAB004 (7.5 ± 0.53 MBq, 11.7 ± 0.82 µg, 100 µL) and then imaged with SPECT-CT (Nanoscan SC, Mediso, Budapest, Hungary) at 4, 24, 48 and 120 h post injection. Following the last imaging timepoint, animals were euthanized (primary: CO_2_ asphyxiation, secondary: cardiac resection) and the following organs resected, weighed and counted for radioactivity content: blood, liver, kidneys, tumors, spleen, pancreas, femur, quadriceps muscle, whole tail, and lungs.

### Image Processing, Image Analysis and Dosimetry

SPECT and CT post-processing and analysis was performed with VivoQuant 3.5 (Invicro, LLC) and resulted in one SPECT-CT image set per animal per timepoint. Detailed image registration and post-processing methods can be found in the Online Data Supplement.

**Figure 1:**
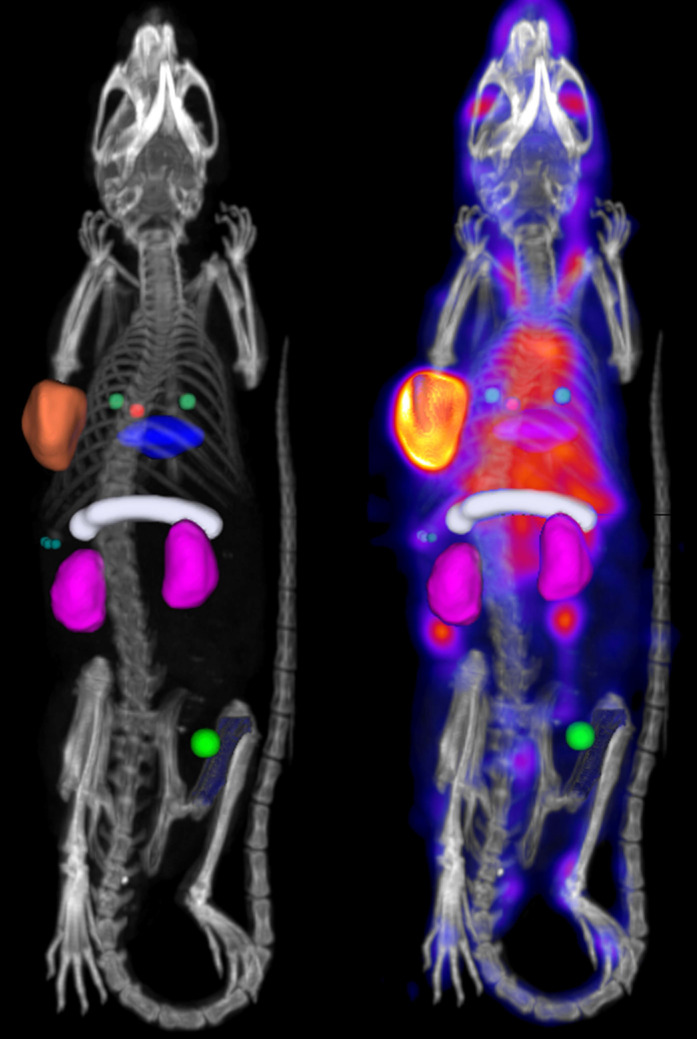
** Processed SPECT-CT image with bed-removal, animal specific cropping and region of interest placement for analysis.** Whole blood (left-ventricle), red sphere. Bone (femur), blue. Kidneys (both), purple. Liver, blue disk. Lungs, two green spheres. Muscle, bright green sphere, Pancreas, white cylinder. Spleen, three small green spheres. Tumor (whole tumor), orange.

*In vivo* biodistribution was quantified for the following organs: whole blood (left-ventricle), bone (femur), kidneys (both), liver, lungs, muscle, pancreas, spleen and tumor (whole tumor). Detailed information on the placement of the regions of interest (ROI) can be found in the Online Data Supplement. Example SPECT-CT images with ROI placement are included in Figure 1. From each ROI, the *in vivo* concentration of ^111^In-DOTA-hTAB004 was quantified assuming a tissue density of water (1 g = 1 mm^3^) and reported as percentage injected dose per gram (%ID/g).

*Ex vivo*, the concentration of ^111^In-DOTA-hTAB004 was calculated as percentage injected dose per gram (%ID/g) and the percentage injected dose (%ID).

The *in vivo* biodistribution data was used to perform several dosimetry estimates using Matlab (Mathworks Inc., Natick MA, USA) and OLINDA/EXM 15. First, dosimetry for ^225^Ac-DOTA-hTAB004 in the mouse was performed to confirm safety of the dose level selected for the therapy study. Detailed methods for the dosimetry calculations are included in the Online Data Supplement. Briefly, the *in vivo* biodistribution data (time activity curves) were used to calculate the mouse mean residence times (MRT) for Indium-111. Mouse MRT values for Indium-111 were transformed to Actinium-225 and input into the OLINDA/EXM 2.0 program (mouse 25 g phantom) 15 for the calculation of the absorbed tissue doses for Actinium-225 in the mouse (RBE of 5). All Actinium-225 daughters were considered to decay in the same organ as the parent. Detailed dosimetry methods are included in the Online Data Supplement.

Next, dosimetry estimates for ^111^In-DOTA-hTAB004 in the human (male and female) were calculated to determine the safety profile of ^111^In-DOTA-hTAB004 compared to two other clinically established Indium-111-labeled antibody imaging agents. Specifically, Capromab pendetide (Capromab pendetide, DTPA chelator, 150 kDa) and ^111^In-J591 (DOTA chelator, 150 kDa) which are both full-length antibodies with similar molecular weights to hTAB004 (150 kDa). Mouse MRT values for Indium-111 were scaled to the human (male and female) and input into the OLINDA/EXM 2.0 program for the calculation of the absorbed tissue doses for Indium-111.

### Therapeutic efficacy

To assess the efficacy of ^225^Ac-DOTA-hTAB004, HCC70 orthotopic tumor bearing nude mice (two groups, n=5 per group) were intravenously injected with either ^225^Ac-DOTA-hTAB004 (18.5 kBq, 12.5 µg, 100 µL) or DOTA-hTAB004 (12.5 µg, 100 µL). Body weights and tumor caliper measurements were recorded three-times per week for 48 days post-dosing. Euthanasia criteria/humane endpoints were; tumor volume >1,500 mm^3^, tumor length >20 mm, weight loss >20% from maximum recorded weight and/or any sign of distress/pain (rough coat, unkept appearance, malaise). Animals were followed for 48 days post dosing or until all mice met a humane endpoint (whichever came first).

### Tumor Volume and Survival Analysis

Tumor volumes were calculated from caliper measurements using the following formula; V = (W^2^ × L)/2 16. In addition, the percentage change in tumor volume from baseline (day -2) was calculated. Statistical analysis was performed on the tumor volumes using the Student's unpaired t-test. The number of days between dosing and the humane or study endpoint was used to assess differences in survival between the ^225^Ac-DOTA-hTAB004 and the DOTA-hTAB004 groups via the Kaplan-Meier curve and the log-rank test. The null hypothesis (H_0_) for the log-rank test stipulates there is no difference in the survival functions between groups ^225^Ac-DOTA-hTAB004 and DOTA-hTAB004. The R statistical computing software was used for all statistical analyses.

Data are reported as mean ± standard deviation unless otherwise stated. A 5% significance level was employed, thus P-values less than 0.05 were identified as statistically significant.

## Results

### Conjugation, Radiolabeling and Stability

The DOTA-to-antibody ratio of the DOTA-hTAB004 was determined to be 4.7 via LC-MS (Figure S1 and Table S2).

The ^111^In-DOTA-hTAB004 production provided a yield >70% (iTLC), radiochemical purity >99% (HPLC-SEC), and specific activity of 844 kBq/µg (22.8 µCi/µg). The production of ^225^Ac-DOTA-hTAB004 provided a yield of >97% (iTLC) and a specific activity of 5 kBq/µg (0.135 µCi/µg). For the full production details see Table S3. Compounds were diluted with DOTA-hTAB004 to achieve the required specific activity for *in vivo* work (Table S3).

The stability assays of ^111^In-DOTA-hTAB004 in formulation (MES buffer, 0.5 M, pH 5.5) at 2-7 ºC and in mouse serum at 37 ºC showed that the radiochemical purity remained >99% over 120 h. The stability assays of ^225^Ac-DOTA-hTAB004 in formulation (MES buffer, 0.5 M, pH 5.5) at 2-7 ºC showed the radiochemical purity remained >99% over 120 h with some decrease in purity in mouse serum between 24 h (99%) and 120 h (82%; Table S4 and Figure S2).

### Affinity and Internalization

**Figure 2:**
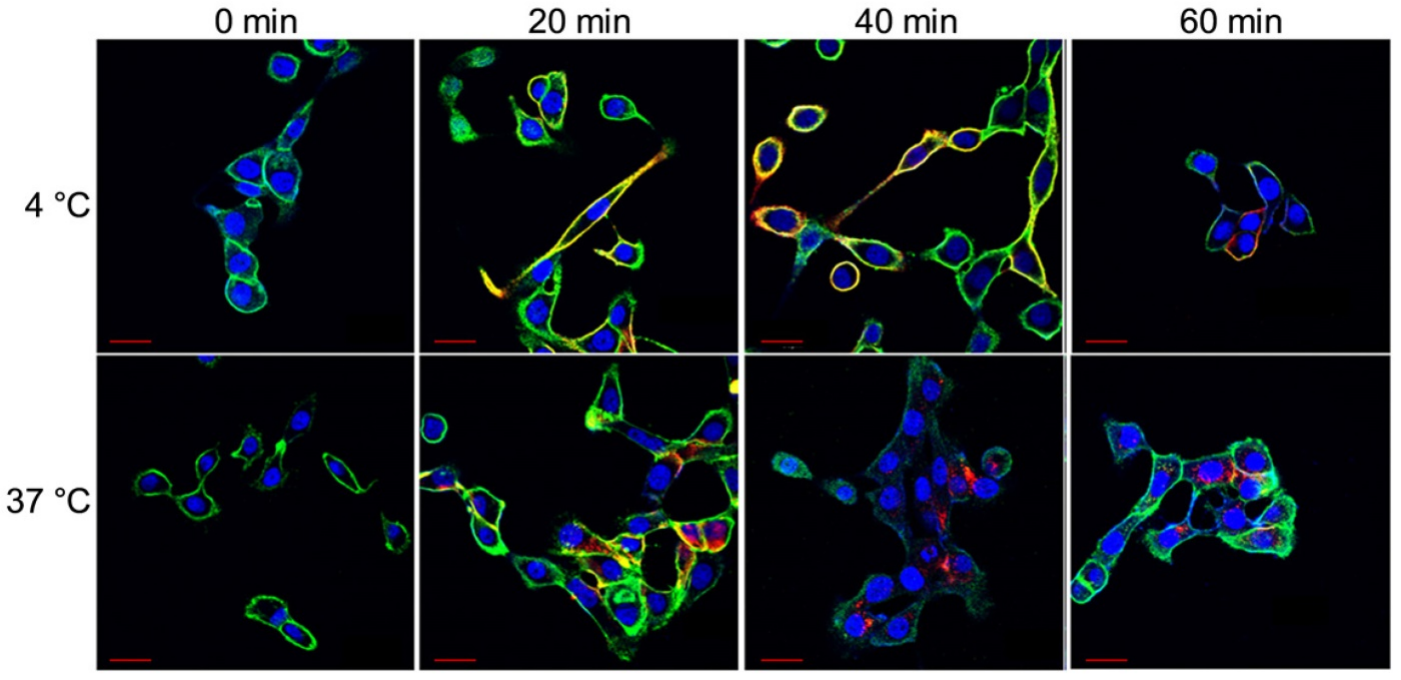
** Representative confocal images of HCC70 cells incubated with HL647-hTAB004 at 4 °C and 37 °C at different time points (0, 20, 40, 60 min).** Blue = DAPI, Green = WGA/cell membrane, Red = HL647-hTAB004. Scale bar, 30 µm. Confocal images for the 4 °C incubation over 60 min shows the HL647-hTAB004 (red) colocalized at the cell membrane (green). In contrast, confocal images for 37 °C over 60 min show the hTAB004 (red) being trafficked intracellularly.

Select confocal images from the hour-long internalization assay is included in Figure 2. The images indicate that at 4 °C HL647-hTAB004 is mostly localized at the cell membrane throughout the course of the study while, at 37 °C, it is almost entirely internalized by 40 min.

ELISA and flow cytometry were used to assess whether the binding of hTAB004 to tMUC1 was impacted by the DOTA conjugation and labeling procedures. The results of the ELISA against KCM lysate and flow cytometry assays indicate that the DOTA conjugates (DOTA-hTAB004, ^115^In-DOTA-hTAB004, and ^139^La-DOTA-hTAB004) have equivalent binding affinity to the hTAB004. ELISA and flow cytometry results are included in the Online Data Supplement (Figure S3).

### Biodistribution of ^111^In-DOTA-hTAB004 in Tumor Bearing Mice

**Figure 3:**
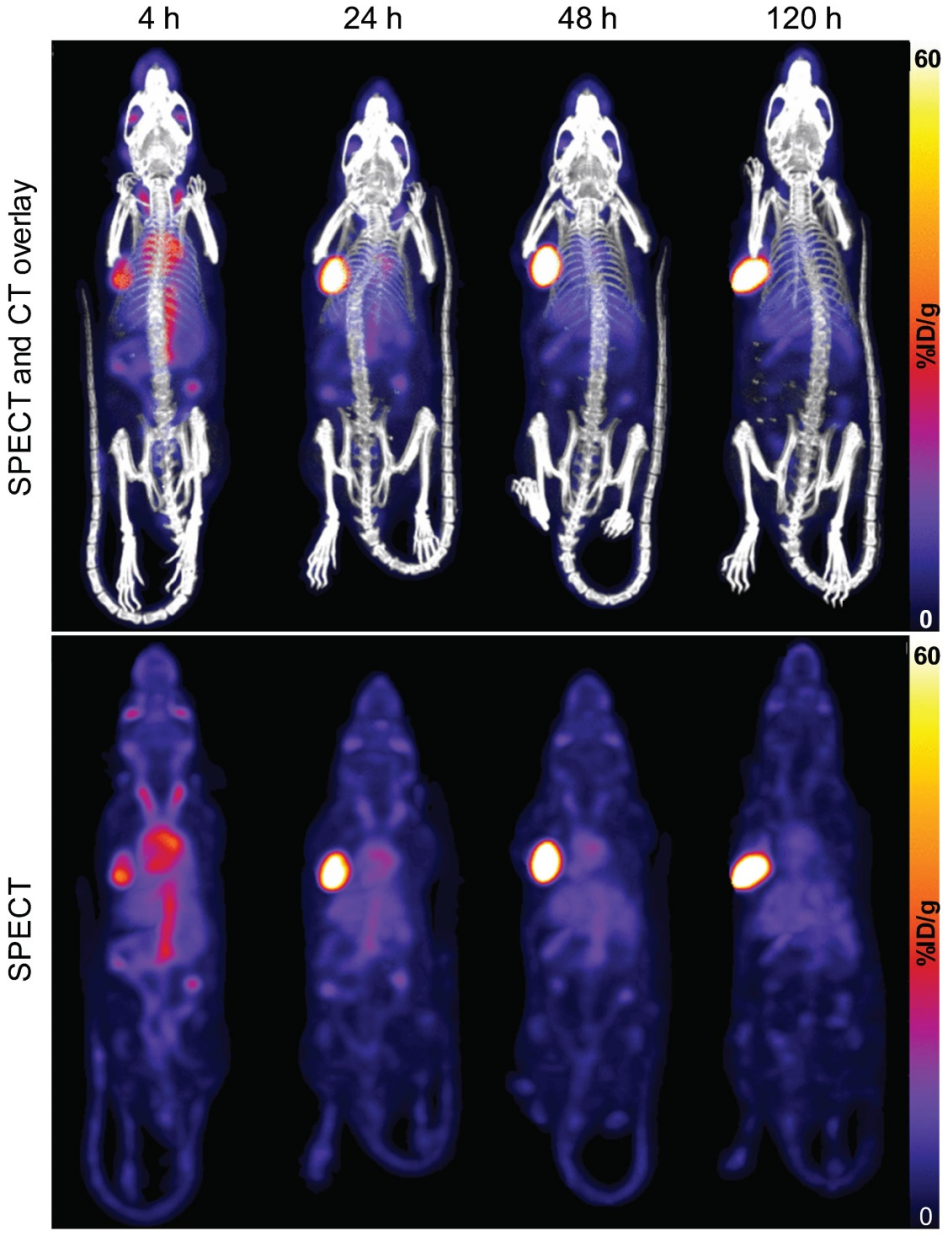
SPECT-CT (top) and SPECT only (bottom) maximum intensity projection images of one representative mouse (ID823) over time (4 - 120 h) show progressive accumulation of ^111^In-DOTA-hTAB004 at the tumor site over 120 h. Color scale is 0-60 %ID/g.

A representative time-series image of the biodistribution of ^111^In-DOTA-hTAB004 over 120 h is included in Figure 3.

**Figure 4:**
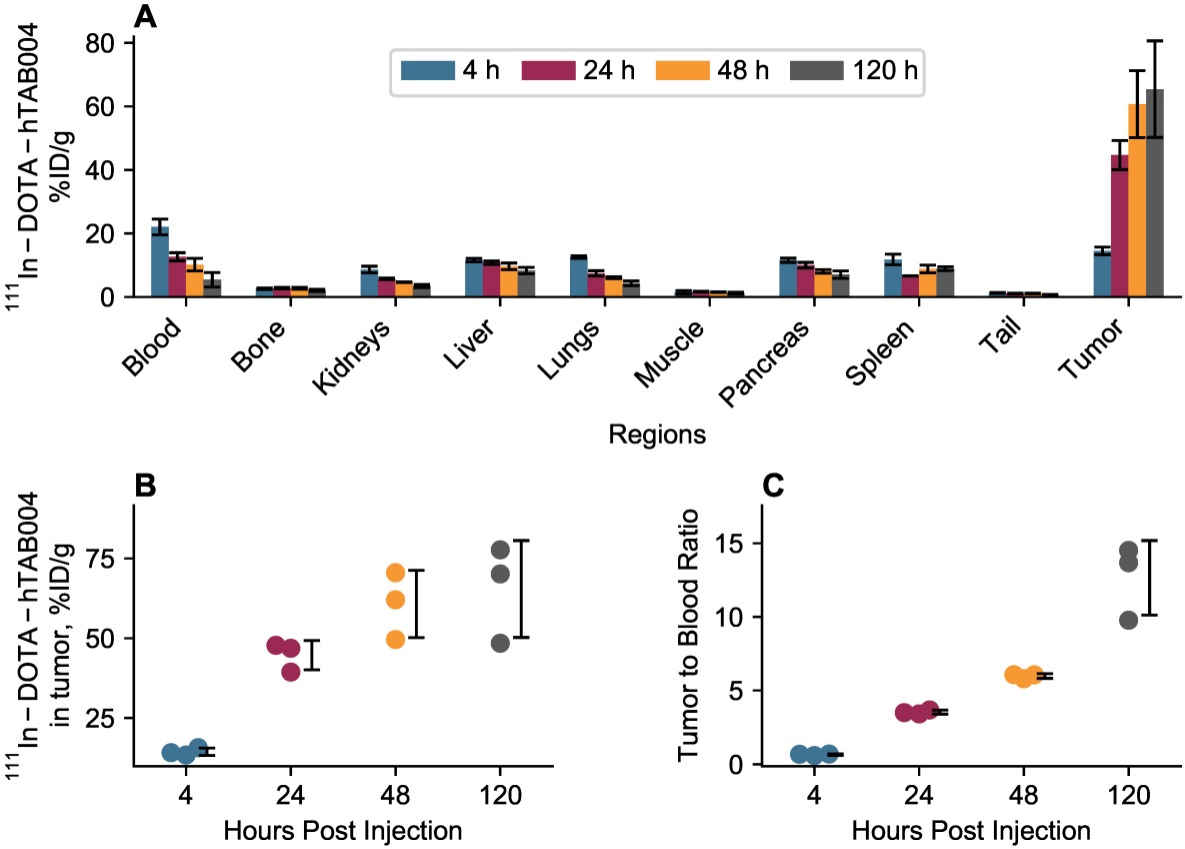
***In vivo* biodistribution of ^111^In-DOTA-hTAB004 in orthotopic HCC70 tumor bearing NSG mice. (A)** ^111^In-DOTA-hTAB004 concentration (%ID/g) vs. time for each organ quantified. The tumor reached a maximum at 120 h with 65.4 ± 15.2 %ID/g. **(B)** Individual tumor ^111^In-DOTA-hTAB004 concentration data. **(C)** Tumor-to-blood ratio over time. At 120 h, the tumor had 12.7 ± 2.5 times more ^111^In-DOTA-hTAB004 than in the blood.

*In vivo* biodistribution results are included in Figure 4. The *in vivo* biodistribution shows that the tumor accumulation of ^111^In-DOTA-hTAB004 increased over 120 h reaching a maximum of 65.4 ± 15.2 %ID/g (11.7 ± 2.4 %ID). All other organs (blood, bone, kidneys, liver, lungs, muscle, pancreas, spleen) had <10% ID/g of ^111^In-DOTA-hTAB004 at 120 h. The tumor-to-blood ratio increased over time, with the tumor having 12.7-fold higher concentration than the blood at 120 h (12.7 ± 2.5 tumor-to-blood ratio at 120 h).

**Figure 5:**
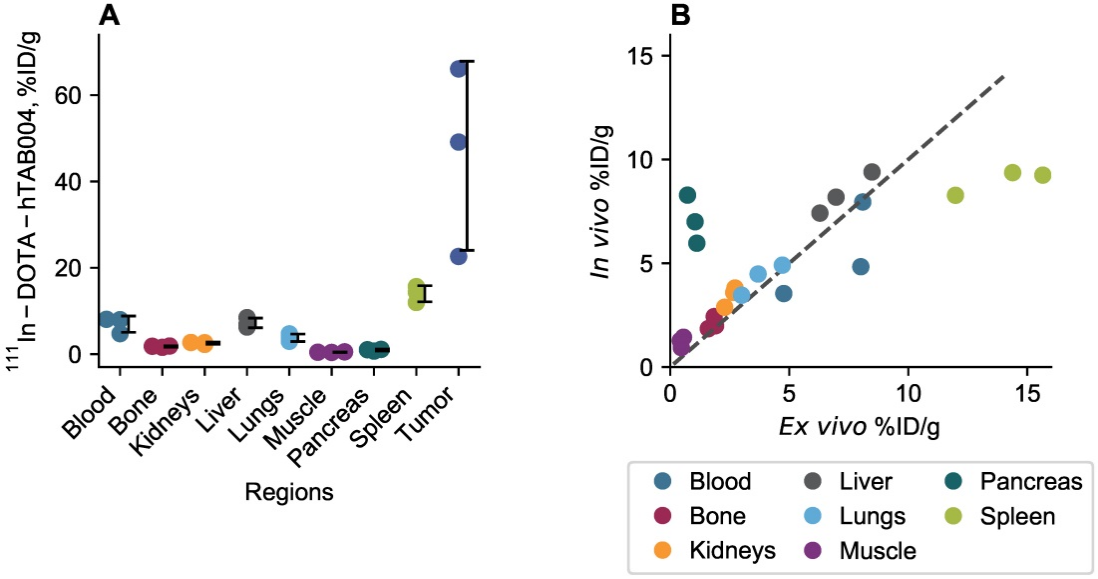
** (A)** *Ex vivo* biodistribution of ^111^In-DOTA-hTAB004 in orthotopic NSG mice at 120 h. **(B)** *Ex vivo* data plotted against the in vivo data at 120 h with the line of identity. *Ex vivo* blood plotted against in vivo left ventricle ROI. In general, there was a good agreement between the in vivo and ex vivo biodistribution data.

*Ex vivo* biodistribution results at 120 h are included in Figure 5. At 120 h the *ex vivo* biodistribution data showed the tumor to have 45.9 ± 21.9 %ID/g (11.3 ± 2.2 %ID) of ^111^In-DOTA-hTAB004. Similarly, the tumor-to-blood ratio at 120 h was 7.1 ± 3.8. The remaining organs (blood, bone, kidneys, liver, lungs, muscle, pancreas, spleen) had <14 %ID/g at 120 h. The *ex vivo* biodistribution results were well-matched to the *in vivo* biodistribution at 120 h excluding the spleen, pancreas, and blood which were not as well matched. The discordant *in vivo* and *ex vivo* results for the spleen and pancreas may be due to the challenges of sampling such small organs via SPECT imaging. The *in vivo* blood activity was sampled via the left ventricle and therefore activity from the lungs may contribute to the higher *in vivo* concentration compared to *ex vivo*.

### Dosimetry of ^225^Ac-DOTA-hTAB004 in mouse and ^111^In-DOTA-hTAB004 in human

The organ specific radiation dosimetry estimates for ^225^Ac-DOTA-hTAB004 in the mouse are included in the Online Data Supplement (Table S5). The dose limiting organ was the spleen with 463.3 ± 32.6 mGy/kBq, followed by the liver (339.3 ± 70.9 mGy/kBq) and lungs (182.2 ± 22 mGy/kBq). The mean absorbed dose for the whole body was found to be 110.2 ± 7.4 mGy/kBq. The mouse ^225^Ac-DOTA-hTAB004 dosimetry estimates were used to determine a safe and therapeutic dose for the efficacy portion of the study. Specifically, we targeted keeping the estimated kidney and spleen dose <10 Gy 17,18 which is well below the 23 Gy limit for nephrotoxicity. In addition, we aimed to keep the skeleton/bone marrow dose below the conventional threshold of <2 Gy 19 and the tumor dose in the target therapeutic range of 30-40 Gy 20. With these considerations an 18.5 kBq dose was selected for the therapy study. This dose was estimated to deliver the target therapeutic dose to a 250 mm^3^ tumor, while maintaining estimated bone marrow, kidney and spleen doses at 1.5, 2.3 and 8.6 Gy, respectively (Table S5).

**Figure 6:**
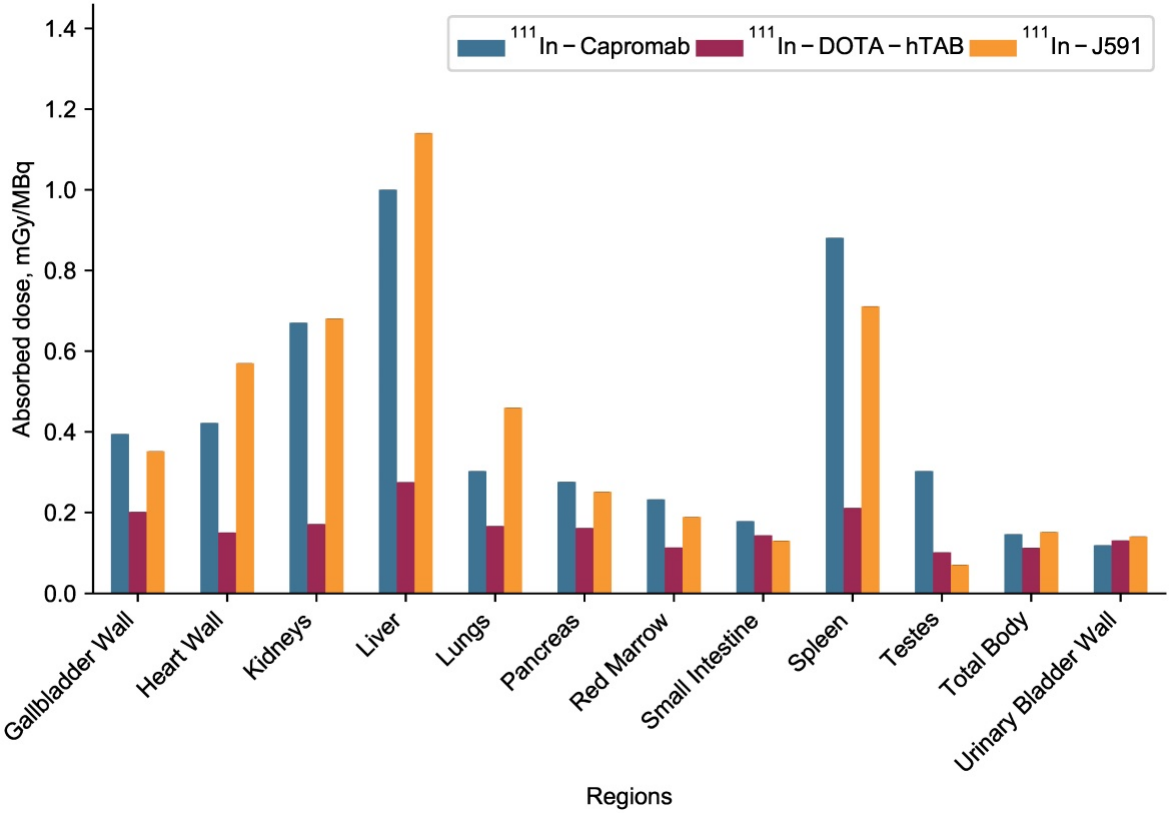
Dosimetry of ^111^In-DOTA-hTAB004 (male) compared to other clinically utilized oncology imaging agents, ^111^In-Capromab and ^111^In-J591.

The complete organ specific radiation dosimetry estimates for ^111^In-DOTA-hTAB004 (mGy/MBq) in the adult human male and female are included in the Online Data Supplement (Table S6). The dosimetry of ^111^In-DOTA-hTAB004 compared to two other clinically established Indium-111-antibodies, ^111^In-Capromab 21 and ^111^In-J591 22, is summarized in Figure 6. Human dosimetry calculations show the liver to be the dose limiting organ (female 0.331 ± 0.0135 mGy/MBq; male 0.275 ± 0.0131 mGy/MBq) followed by the spleen. The mean effective dose for females was found to be 0.172 ± 0.003 mSv/MBq, and that for males was 0.129 ± 0.001 mSv/MBq (Table S6).

### Efficacy of ^225^Ac-DOTA-hTAB004

All animals in the control group were euthanized by study day 34, therefore all inter-group statistical comparisons were performed on data collected up to and including day 34. Intra-group comparisons utillised all data points. Changes in body weight (%) from the day of dosing are included in Figure S4.

**Figure 7:**
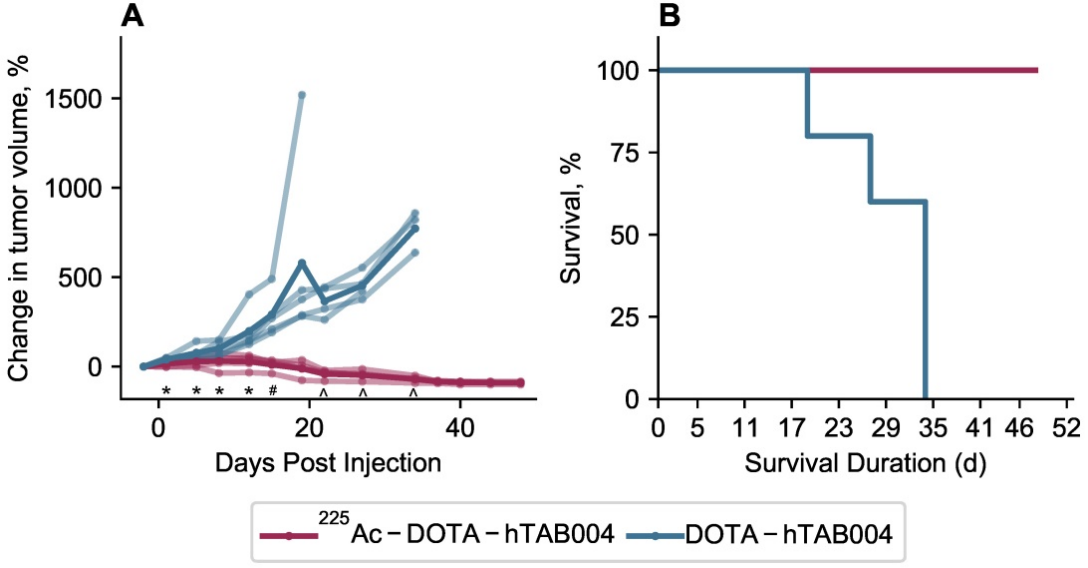
** (A)** Tumor volume in animals administered with ^225^Ac-DOTA-hTAB004 (red) compared to control animals administered DOTA-hTAB004 (blue) as monitored for the duration of the study. Intra-group comparisons of percentage change in tumor volume at each study day; * P<0.05, ^#^ P<0.01, ^ P<0.001. **(B)** Kaplan-Meier survival curves showing 100% survival in treatment group until the end of the study (D=48; red) and 0% survival in control group (blue).

Statistical analysis of the tumor volumes indicate that the ^225^Ac-DOTA-hTAB004 group had significantly lower tumor growth than the DOTA-hTAB004 control group at days 1, 5, 8, 12, 15, 22, 27 and 34 (See Figure 7 and Table S7). Furthermore, the control group had significantly increased tumor volumes, compared to baseline from day 1 out to day 34. In contrast, the treatment group had no significant change in tumor volume from baseline to day 27 (no tumor growth). From day 34 to day 48, the treatment group had a significant decrease in tumor volume compared to baseline (P<0.05).

To assess differences in survival (days on study) between the two groups, the log-rank test was used (Table 1). For the analysis, one animal was euthanized for a non-study-specific endpoint and was therefore censored in the survival analysis (^225^Ac-DOTA-hTAB004 group, day 12). In addition, all remaining animals in the treatment group were defined as surviving till day 48 when they were euthanized at the end of the study. The results of the log-rank test showed that treatment with ^225^Ac-DOTA-hTAB004 was associated with significantly longer survival (P=0.007). The Kaplan Meier plot for both groups is included in Figure 7.

## Discussion

The aim of this study was to evaluate the hTAB004 antibody as a theranostic agent for tMUC1-positive TNBC. As part of this work we have shown that both the affinity of hTAB004 to the target antigen and internalization characteristics were maintained following humanization of the antibody. We successfully radiolabeled the DOTA-hTAB004 with both Indium-111 and Actinium-225 using a mild (pH 5.5, 37 °C), single step process resulting in high radiochemical purity. In addition, the labeled hTAB004 derivatives (DOTA-hTAB004, ^115^In-DOTA-hTAB004 and ^139^La-DOTA-hTAB004) were shown to maintain their antigen binding specificity. Our *in vivo* studies in HCC70 orthotopic tumor xenograft bearing nude mice demonstrated both high tumor concentrations (65.4 ± 15.2 %ID/g) and high tumor-to-blood ratios (12.7 ± 2.5) for ^111^In-DOTA-hTAB004 at 120 h. In addition, a single 18.5 kBq administration of ^225^Ac-DOTA-hTAB004 increased survival (P<0.007) and resulted in consistently lower tumor volumes compared to the control group after 12 days (P=0.01) while avoiding any severe adverse effects. Lastly, dosimetry estimates extrapolated to humans were favorable compared to two other clinically available Indium-111-antibody imaging agents. Taken together, our results provide convincing proof-of-concept support for hTAB004 as a theranostic agent in triple negative breast cancer.

There are several key steps to developing a theranostic agent using the radio-metal combination of Indium-111 and Actinium-225. First, radiochemistry methods must be developed for the relevant isotopes which obtain acceptable yields while maintaining the antibody's stability and antigen affinity. In addition to developing radiochemistry methods, the antibody must be able to tolerate the conditions required for chelation and radiolabeling and afterwards retain its ability to bind the target antigen. We evaluated the tMUC1 binding characteristics of DOTA-hTAB004, ^115^In-DOTA-hTAB004, and ^139^La-DOTA-hTAB004 via ELISA and flow cytometry and found no significant changes.

The stability of the radiolabeled antibody in formulation and serum is also key to its future success as a theranostic. The stability of the ^111^In-DOTA-hTAB004 and ^225^Ac-DOTA-hTAB004 derivative were assessed and found to be highly stable (>99% RCP) for up to 120 h in formulation and in mouse serum for 24 h. By confirming the functionality (antigen-binding) and serum stability of the labeled antibody we were able to de-risk the subsequent proof of concept *in vivo* animal studies.

The second key step for developing a successful theranostic agent utilizing Actinium-225 is for the antibody to target the antigen and subsequently internalize. Actinium-225 is an alpha particle emitting radionuclide that yields several daughter radionuclides and a net of four alpha particle emissions in its decay scheme. Given that each alpha particle can deposit 5 ± 8 MeV in a short ionizing track of 50 ± 80 µm (2 ± 4 typical cell diameters) 23 alpha particles are lethal to the cells that are targeted while sparing most normal surrounding cells. Radiohalogens will redistribute from the tumor throughout the body after internalization unless they are chemically engineered to be retained 24. Radiometals typically remain inside a cell when internalized, despite cellular catabolic events, thus significantly reducing radiation exposure to non-target tissues. When utilizing Actinium-225, given the 10 day half-life, recoil energy and complex decay chain it should be bound to an efficiently internalizing antibody to ensure all the radioactive daughters will remain internalized in target cells 25. We demonstrated the internalization of the tMUC1 antigen in the HCC70 cell line via fluorescent microscopy. In addition to the *in vitro* internalization assays, the *in vivo* biodistribution demonstrated high tumor targeting with >60 %ID/g in the tumor at 120 h post injection indicating that the ^111^In-DOTA-hTAB004 imaging agent is indeed able to effectively target the antigen.

The third step for the development of a successful theranostic is ensuring that the agent is safe. For safety and efficacy of the theranostic, it must accumulate in biologically meaningful amounts within tumors and have low off-target organ accumulation. For the hTAB004, we have demonstrated that the *in vivo* tumor accumulation of ^111^In-DOTA-hTAB004 was high (>60% ID/g) and the accumulation in the key organs (kidney, liver, spleen) was low (<10 %ID/g). These *in vivo* biodistribution results led to favorable dosimetry for ^111^In-DOTA-hTAB004 compared to two other oncology imaging agents (^111^In-Capromab 21 and ^111^In-J591 22). It follows that the dosimetry profile for ^111^In-DOTA-hTAB004 is favorable for clinical translation. In addition, no agent-related mortality or morbidity occurred although there was a decrease in body weight of the ^225^Ac-DOTA-hTAB004 treated mice over 48 days.

For alpha-emitting theranostics, the off-target organ dosimetry is very important for later patient safety. Therefore, it is imperative that the first step be visualizing the target (biodistribution with ^111^In-DOTA-hTAB004), followed by individualized dosimetry of the alpha-emitting therapeutic agent (dosimetry of ^225^Ac-DOTA-hTAB004) to establish the safety profile and appropriate therapeutic dosing. We utilized this approach to determine a safe and therapeutic dose of 18.5 kBq in the pre-clinical model. This personalized medicine approach allows the selection of patients that express the target, and hence will benefit from the treatment. Further, the safety profile of treatment can be significantly enhanced by minimizing off-target effects of Actinium-225 to normal tissue using individualized dosimetry.

The final step in theranostic development is that the theranostic must perform the ultimate goal of causing tumor cell death and, ultimately, cancer remission. Tumor bearing nude mice treated with ^225^Ac-DOTA-hTAB004 showed clear response 40 days following therapy (tumor volume ≥ 90% decrease from baseline). In contrast, by day 34 all controls had been euthanized. It follows, that ^225^Ac-DOTA-hTAB004 significantly increased survival and therefore these results strongly support the utility of ^225^Ac-DOTA-hTAB004 as a therapeutic in TNBC.

One of the key considerations in alpha-emitters as theranostics is the possibility that heterogeneity of antigen expression in tumors combined with the short ionizing track of alpha emitters may limit the overall efficacy of alpha emitters. Specifically, regions of tumor that lack the expression of the target antigen will not receive the ionizing radiation from alpha-emitters compared to beta emitters. Despite the localized release of ionizing energy with alpha emitters there are several mechanisms by which they are able to cause widespread cell death in tumors, even in regions without antigen expression. These processes include the radiation-induced bystander effect and the abscopal effect 23. These processes describe how cells, near or far from the tumor, can be damaged and killed even though they were not directly exposure to ionizing radiation. These secondary effects in combination with the direct double strand DNA break and severe chromosomal damage of alpha particle emitters may explain the absolute tumor regression we report in this study.

### Limitations

There are several limitations to these proof-of-concept studies. First, the specificity of the tumor accumulation of ^111^In-DOTA-hTAB004 was not confirmed by the inclusion of a blocking group (^111^In-DOTA-hTAB004 co-injected with an excess of hTAB004) nor an isotype control group. Therefore, we cannot exclude the possibility that a portion of the tumor accumulation of ^111^In-DOTA-hTAB004 was due to vascular leakage and/or non-specific binding. *Ex vivo* imaging data collected with ^124^I-TAB004 had very low retention in the tumor (~5 %ID/g at 48 h, data not shown) suggesting that non-specific binding is not a major driver of retention in this tumor model. However, these results should be confirmed with *in vivo* studies as a next step. Second, as hTAB004 doesn't cross react with murine MUC1, we may be underestimating the accumulation of the antibody in healthy organs. Future work is planned with a human MUC1 transgenic (C57BL/6-MUC1.Tg) mouse model 26 which would serve as a better predictor of human distribution. Lastly, in a series of elegant experiments Sharma et. al. 27 recently reported that there are Fc mediated differences in biodistribution of antibodies in different strains of immunocompromised mice. Specifically, that NSG and NOD SCID mice have higher spleen, liver and bone concentrations compared to Nu/Nu mice. Our utilization of NSG mice to perform the imaging and dosimetry calculation may have resulted in overestimation of spleen uptake of the imaging agent. In addition, Sharma et, al. report that tumor concentrations tend to be higher in Nu/Nu mice compared to NSG mice. Therefore, it is plausible that the tumor concentration of ^225^Ac-DOTA-hTAB004 in nude mice was greater than we predicted from the biodistribution of ^111^In-DOTA-hTAB004 in NSG mice.

An additional limitation is that we did not perform direct comparisons of the biodistributions of ^111^In-DOTA-hTAB004 and ^225^Ac-DOTA-hTAB004 to assess bioequivalence. From studies performed previously it is documented that changing the radiometal alone (with no other changes to the chelate or molecule) can cause some differences in the *in vivo* biodistribution of a molecule 22,28. This result is likely due to a combination of different radiometal coordination chemistry as well as different *in vivo* processing of the metabolized radioisotope 29. There is currently no non-radioactive isotopologue of Actinium-225, making its coordination chemistry difficult to investigate; in addition, there is currently no imaging isotopologue of Actinium-225 with favorable half-life and decay emissions.

Additional studies are warranted to further characterize the hTAB004 antibody, including; additional pharmacokinetic, *in vitro* binding and immunoreactivity assays, and also biodistribution and efficacy studies with additional control groups (blocking, untreated) and larger group N.

### Clinical implications

Recently, a phase 3 trial of another MUC1 antibody, hPAM4 conjugated to Yttrium-90 was used in combination with gemcitabine for the treatment of metastatic pancreatic ductal adenocarcinoma (PDA) 30. Due to no benefit in overall survival between placebo+gemcitabine and ^90^Y-hPAM4+gemcitabine, the trial was terminated in early 2019. It is possible that the trial failed because metastatic unresectable PDA is a highly desmoplastic and fibrotic disease (and hence poorly vascularized and accessible) and is resistant to most treatment regimens 31. Our work is focused on a very different disease in TNBC. Although we have not done a back-to-back comparison with hPAM4 antibody (which is a PDA-specific MUC1 antibody), our data clearly suggests that hTAB004 is highly specific for TNBC, internalizes efficiently and therefore we envision that ^111^In/^225^Ac-hTAB004 can be efficacious in a TNBC setting.

Epidermal growth factor receptor (EGFR) is another promising target for TNBC 32, however, only ~40% of patients express the receptor. In comparison, tMUC1 is expressed on >90% of TNBC patients and thus remains a superior target for TNBC. Furthermore, monotherapy with ^177^Lu-anti-EGFR antibody was not effective in the MDA-MB-231 murine model of TNBC. A combination of Docetaxel+doxorubicin+PARP inhibitor rucaparib+^177^Lu-anti-EGFR anitbody had to be used for the therapy to be effective 32. Based on our data, treatment with ^225^Ac-tMUC1 antibody clearly suggests a better treatment option even as a monotherapy.

Actinium-225 labeled antibodies are currently being used in several clinical trials as oncology therapeutics 33. Short term side effects of Actinium-225 labeled agents include acute hematologic toxicity and xerostomia 34. The long-term side effects are not yet well established but one of the major concerns is the potential for long-term blood toxicities and renal damage 35,36. Radiation damage to the kidneys is a particular area of interest. Circulating progeny, particularly Bismuth-213, have a propensity to be captured by the kidneys 37. In addition, the kidneys are particularly sensitive to radiation 38. Future ^225^Ac-DOTA-hTAB004 toxicity studies will need to evaluate both the the short-term and possible long-term blood and renal damage following treatment to ensure safety prior to translation into humans.

## Conclusion

We investigated hTAB004 as a theranostic candidate for triple negative breast cancer. As part of our evaluation we developed a robust radiolabeling method for producing both ^111^In-DOTA-hTAB004 and ^225^Ac-DOTA-hTAB004 while achieving high radiolabeling efficiency, purity and stability. *In vitro* assays demonstrated tMUC1 antigen engagement and internalization of hTAB004; key factors that enable Actinium-225 radiolabeled agents to be efficacious. *In vivo* studies allowed us to quantify the biodistribution and calculate the dosimetry of ^111^In-DOTA-hTAB004 showing satisfactory tumor accumulation and a clinically relevant dosimetry profile compared to other ^111^In-labeled antibodies. Lastly, our *in vivo* therapeutic study showed tumor regression and a significant improvement in survival in treated mice over control mice. Taken together, we have presented evidence that hTAB004 is a promising theranostic agent for TNBC. Additional pre-clinical studies, including long-term toxicity studies, are warranted to progress hTAB004 towards the clinic.

## Supplementary Material

Supplementary figures and tables.

## Figures and Tables

**Table 1:** Survival Statistics; Log-Rank Test

Group	N	Observed	Expected	(O-E)^2^/E	(O-E)^2^/V
^225^Ac-DOTA-hTAB004	5	4	6.66	1.06	7.16
DOTA-hTAB004	5	5	2.34	3.02	7.16

χ^2^ = 7.2 on 1 degree of freedom, P=0.007.

## Abbreviations

^111^In: Indium-111
^177^Lu: Lutetium-177
^209^Pb: Lead-209
^209^Ti: Titanium-209
^213^Bi: Bismuth-213
^213^Po: Polonium-213
^217^At: Astatine-217
^221^Fr: Francium-221
^225^Ac: Actinium-225
CT: computed tomography
DOTA: 1,4,7,10-tetraazacyclododecane-1,4,7,10-tetraacetic acid
EDTA: ethylenediamine tetraacetic acid
FBS: fetal bovine serum
HEPES: 4-(2-Hydroxyethyl)-1-piperazineethanesulfonic acid
HER2/neu: human epidermal growth factor receptor 2/neural tumor
HPLC: high performance liquid chromatography
TAB004: murine antibody to transformed mucin 1
hTAB004: humanized murine antibody to transformed mucin 1
MUC1: mucin 1
tMUC1: transformed mucin 1
PBS: phosphate buffered saline
PSMA: prostate specific membrane antigen
RBE: relative biological effectiveness
RCP: radio-chemical purity
SA: specific activity
SEC: size exclusion chromatography
SPECT: single photon emission computed tomography
TNBC: triple-negative breast cancer
TRT: targeted radiotherapy
