# Supplementary material for: Preclinical evaluation of an 111In/225Ac theranostic targeting transformed MUC1 for triple negative breast cancer
Source: Theranostics. 2020 May 25;10(15):6946–58. doi: 10.7150/thno.38236 (PMC7295045; doi:10.7150/thno.38236)
Supplement: Supplementary file 1 — Supplementary figures and tables. [file thnov10p6946s1.pdf]

Online Data Supplement
Methods

Liquid chromatography–mass spectrometry

The hTAB004 and DOTA-hTAB004 were analyzed via LC-MS (Table S1) with deglycosylation. High charge states were observed. The mass data was deconvoluted to obtain the mass of the intact DOTA-hTAB004. Use of PNGase to remove glycan changes simplified the analysis by allowing separation of clusters of species. Areas under the deconvoluted peaks were used to calculate a CAR value.

Table S1: HPLC parameters

<table>
<tr><td>System</td><td>Agilent 1290 with 6520 TOF</td><td rowspan="6">Sample prep:

0.2 µL PNGase added to 10 µL mAb. 0.2 µL PNGase added to 5 µL conjugate. Solutions were incubated at 37 °C overnight. Solutions were then diluted with 100 µL 1% Formic acid in water.</td></tr>
<tr><td>Temp.</td><td>80 °C</td></tr>
<tr><td>Flow</td><td>0.5 mL/min</td></tr>
<tr><td>Column</td><td>Agilent PLRP-S, 2.1x50 mm, 3 µm</td></tr>
<tr><td>Mobile phase</td><td>0-2 min 10/90
2-10 min ramp to 90/10
A=0.1% Formic acid in CH_3CN
B=0.1% Formic acid in water</td></tr>
<tr><td>Sample Volume</td><td>10 µL</td></tr>
</table>

In vitro Studies

Sandwich ELISA

ELISA well plates (96-well) were coated with hTAB004, DOTA-hTAB004, ^{115}In-DOTA-hTAB004 and ^{139}La-DOTA-hTAB004 at 3 µg/mL for 24 h at 2-7 °C. Well plates were then washed four times with 1x wash buffer, blocked with 10% FBS in PBS for 1 h, and then again washed four times with 1x wash buffer. KCM lysate (KCM is a murine pancreatic cancer cell line that expresses human tMUC1 generated and maintained in-house) was tested against each antibody (100 µL) in triplicate at the following concentrations of 0, 0.625, 1.25, 2.5, 5. and 10 µg/mL. Stock solutions of Liquichek Tumor Marker Control Level 1 and Level 2 (BioRad, Hercules, CA, USA) were used as samples at a 1:7 dilution. The well-plates were incubated at room temperature for 2 h, washed four times with 1x wash buffer, 100 µL of TAB004-HRP was added and then the plates were incubated at room temperature for 1 h. Plates were washed 4x with 1x wash buffer and 100 µL TMB substrate (TMBW010001, Surmodics, Eden Prairie, MN, USA) added and incubated at room temperature for 30 min, followed by stop solution (TMBW010001, Surmodics, Eden Prairie, MN, USA) and the plate read at 450 nm.

Cell culture and animal inoculations.

HCC70 cells were purchased from ATCC (ATCC CRL-2315, Manassas, VA, USA). Cells were cultured in complete cell media consisting of RPMI-1640 (ATCC 30-2001, Manassas, VA, USA) and 10% FBS (A3160501, Fisher Scientific, Pittsburgh, PA, USA). NSG (N=5) and athymic nude mice (N=15, 005557 and 002019, Jackson Labs, Bar Harbor, ME, USA) were injected with 9×10^6 HCC70 cells suspended in 100 µL of 50/50 mixture of PBS and GFR Matrigel (356230, Corning, Corning, NY, USA) on the animal's left side of either mammary fat pad pair 2 or 3.

Image registration, processing and regions of interest definitions`

The CT was registered to the SPECT image which was then reconstructed with CT based attenuation correction. Reconstructed SPECT images were generated in units of µCi and resampled to 0.2 mm^3 voxels and cropped to a uniform size prior to analysis. CT images were used as an anatomical reference. Bed removal was performed (removal of three-mouse hotel) and SPECT-CT registered images cropped to generate one image set per animal per timepoint.

Regions of interest (ROIs) for the kidneys (left and right) and liver were defined by placing fixed volume phantoms based on the CT and SPECT. The left ventricle (ROI for whole blood), muscle, pancreas and spleen were sampled by placing one, two, and three fixed volume spheres in the anatomically correct positions based on the CT scan. The tumor was hand-segmented by a trained imaging scientist based on the CT image, and with confirmation using the SPECT image. The bone was sampled by generating a region encompassing the femur and thresholding the images based on CT density to remove soft tissue.

Dosimetry Calculations

Several dosimetry estimates were performed. First, dosimetry for ^{225}Ac-DOTA-hTAB004 in the mouse was performed to confirm safety of the dose levels selected for the therapy study. Second, dosimetry estimates for ^{111}In-DOTA-hTAB004 for the human (male and female) were calculated to determine the safety profile of ^{111}In-DOTA-hTAB004 compared to other clinically approved Indium-111-antibody imaging agents.

To estimate dosimetry for ^{225}Ac-DOTA-hTAB004 in the mice, *in vivo* ^{111}In-DOTA-hTAB004 biodistribution time activity curves were translated from ^{111}In-DOTA-hTAB004 to ^{225}Ac-DOTA-hTAB004. Specifically, decay associated with Indium-111 was removed from time activity curves, leaving curves that only account for clearance. Curves were then corrected for Actinium-225 decay at each imaging time point. Mean residence times (MRT) were then calculated for all source organs: kidneys, liver, lungs, and spleen. MRT values were calculated as the area under the curve of the average fraction of injected over time (average of n=3 mice).

MRT values were entered into OLINDA/EXM 2.0 software [1] to calculate absorbed dose values for the 25 g mouse model. All daughter decays of Actinium-225 were considered to decay within the same organ in calculations (Fr-221, At-217, Bi-213, Po-213, Ti-209, Pb-209). A relative biological effectiveness (RBE) value of 5 was used in all Actinium-225 dosimetry calculations for Sievert. To estimate tumor dosimetry the sphere model in OLINDA 2.0 was utilized. The sphere model approximates the self-dose to a unit density sphere, with no contributions from other

source organs and no contribution from this organ to other target organs. A sphere with a mass of 0.1 g and 0.5 g was used for calculations (both results reported).

Human ^{111}In-DOTA-hTAB004 dosimetry estimates were calculated from the *in vivo* biodistribution data (time activity curves). MRT values were determined by calculating the area under the curve of the average fraction of injected activity over time (average of n=3 mice) for all source organs: kidneys, liver, lungs and spleen. Human MRT values were computed from mouse MRT values by an equivalent of the percentage kilogram per gram method [2]. The human organ weight to human weight ratios were determined from the adult male and adult female phantom organ and total body weights from the OLINDA/EXM 2.0 program [1]. The absorbed tissue doses in humans were calculated with the OLINDA/EXM 2.0 software program using the adult male and adult female phantoms with the male and female data, respectively.

Results

Mass spectrometry

The CAR of the DOTA-hTAB004 was determined to be 4.7. The deconvoluted mass spectrometry results are included in Figure S1 and Table S2.

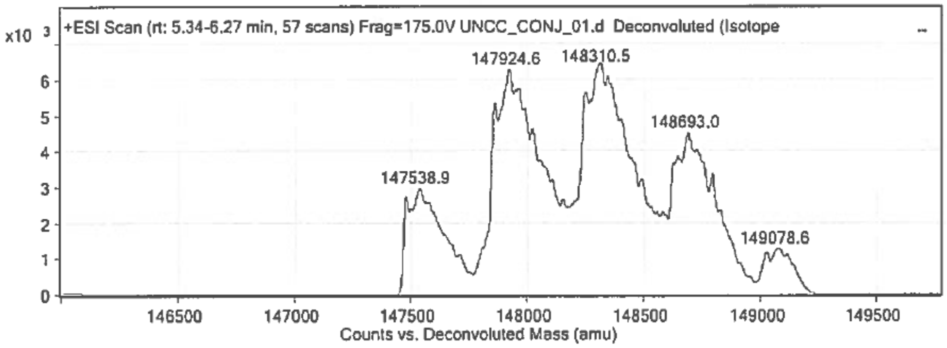

Figure S1: Mass spectrometry data, deconvoluted.

Table S2: Mass spectrometry results

	Chelates / hTAB004	***Area***
**Deglycosylated mass**<br>146,316 g/mol<br><br>**Chelate**<br>DOTA-NHS ester<br><br>**Net mass gain per chelate**<br>386 g/mol	*0*	
	1	
	2	
	3	*561,730*
	4	*1,532,384*
	5	*1,709,787*
	6	*981,575*
	7	*189,131*
	8	
	9	
	10	
	**Total Area**	4,974,607
	**CAR value**	4.7

Radiolabeling and Stability

A summary of the radiolabeling conditions and results are included in Table S3. The ^{111}In-DOTA-hTAB004 and ^{225}Ac-DOTA-hTAB004 stability results in formulation and mouse serum are included in Table S4. In addition, example stability HPLC-SEC plots for ^{225}Ac-DOTA-hTAB004 that were generated by gamma counting 60 s fractions of the product from the column are included in Figure S2.

Table S3: Radiolabeling results

Crude reaction	^{111}In-DOTA-hTAB004	^{225}Ac-DOTA-hTAB004
DOTA-hTAB004	10 µL, 46 µg	20 µL, 92 µg,
Indium-111 chloride or Actinium-225 solid nitrate	45 µL, 55.5 MBq	1.5 µL, 0.47 MBq
MES buffer, 0.5M pH 5.53	450 µL	100 µL
Incubation	60 min at 37 °C	120 min at 37 °C
Radiochemical yield (iTLC)	70%	97%
Radiochemical purity (HPLC-SEC)	>99%	Not assessed
Specific activity	844 kBq/µg	5 kBq/µg

Table S4: Stability in formulation and Mouse serum.

		0 h	2 h	24 h	48 h	120 h
^{111}In-DOTA-hTAB004	Mouse Serum, 37 °C	>99%	>99%	>99%	>99%	>99%
	Formulation, 2-7 °C	>99%	>99%	>99%	>99%	>99%
^{225}Ac-DOTA-hTAB004	Mouse Serum, 37 °C	>99%	-	>99%	-	82%
	Formulation, 2-7 °C	>99%	-	>99%	-	>99%

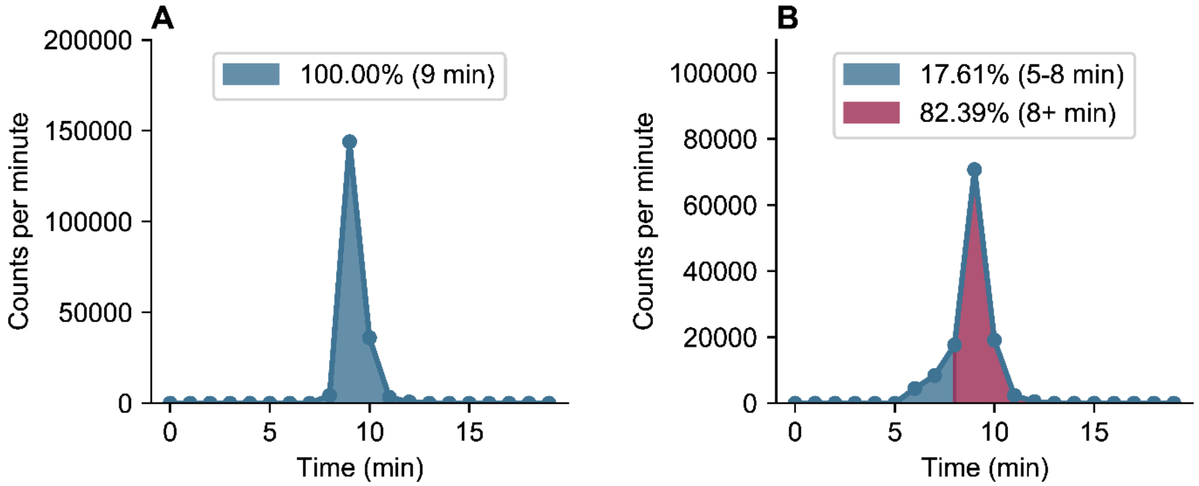

Figure S2: HPLC-SEC radio trace recreated from gamma counting 60 sec fractions. X-axis: Retention time (minutes). Y-Axis: Counts per minute (CPM). Left: Formulation stability (2-7 °C) sample at 120 h. Right: Mouse serum stability (37 °C) sample at 120 h, some broadening of the peak occurred.

Binding affinity of labeled conjugates.

Binding affinity curves are included in Figure S3.

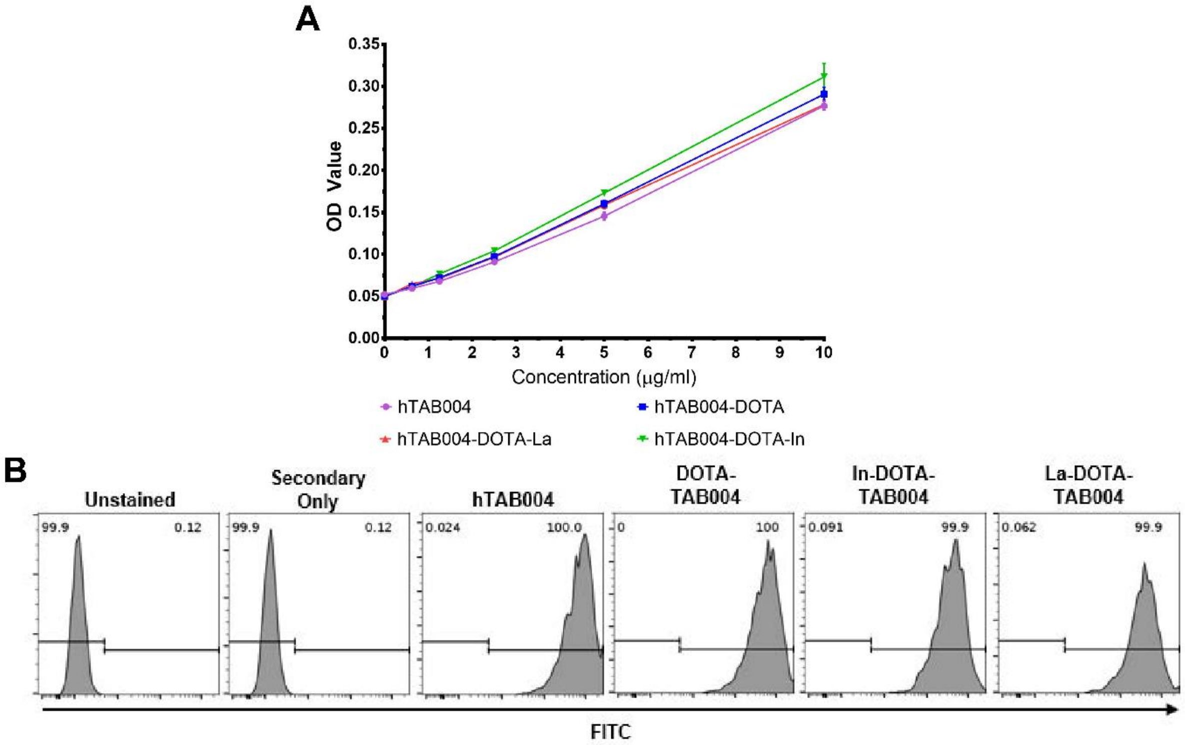

Figure S3. Binding profiles of hTAB004 and hTAB004 conjugates. A) OD values from ELISA of different concentrations of KCM lysate against plated hTAB004 and hTAB004 conjugates. B) HCC70 cells stained with 0.2 µg hTAB004 alone or hTAB004 conjugates. FITC anti-human IgG Fc secondary antibody was used to capture the hTAB004 signal.

^{225}Ac-DOTA-hTAB004 dosimetry for the mouse.

The organ specific absorbed dose estimates for ^{225}Ac-DOTA-hTAB004 in the mouse are included in Table S5.

Table S5: ^{225}Ac-DOTA-hTAB004 absorbed dose estimates in the mouse.

	Absorbed Dose	
**Organ**	**mGy/kBq**	**Gy/18.5 kBq**
Brain	81.3 ± 11.4	1.50
Large Intestine	81.4 ± 11.3	1.51
Small Intestine	81.5 ± 11.3	1.51
Stomach Wall	81.5 ± 11.3	1.51
Heart	81.5 ± 11.3	1.51
Kidneys	125.6 ± 14.1	2.32 *
Liver	339.3 ± 70.9	6.28
Lungs	182.2 ± 22	3.37
Pancreas	81.5 ± 11.3	1.51
Skeleton	82.2 ± 11.5	1.52 #
Spleen	463.3 ± 32.6	8.57
Testes	81.3 ± 11.4	1.50
Thyroid	81.4 ± 11.3	1.51
Tumor (0.1 g)	5,986.7 ± 1,293.7	110.75
Tumor (0.5 g)	1,191.3 ± 258.2	22.04
Urinary Bladder	81.3 ± 11.4	1.50
Total Body	110.2 ± 7.4	2.04

Bone marrow absorbed dose should be < 2 Gy to limit hematological toxicity (Skeleton used as bone marrow surrogate). Relative biological effectiveness of alpha particle was 5.

Tumor dosimetry estimated using both the 0.1 g sphere model and a 0.5 g sphere model. Mean tumor volume on day -2 was 252 mm^3 in the ^{225}Ac-DOTA-hTAB004 and 247.9 mm^3 in the DOTA-hTAB004 group.

* Kidney absorbed dose should be <23 Gy to limit nephrotoxicity.

^{111}In-DOTA-hTAB004 dosimetry in human.

Table S6: ^{111}In-DOTA-hTAB004 dosimetry estimates (mGy/MBq) for adult human male and female.

Absorbed Dose, mGy/MBq	Male	Female
**Target Organ**	***Mean ± SD***	***Mean ± SD***
Adrenals	0.183 ± 0.0021	0.231 ± 0.0017
Brain	0.094 ± 0.0008	0.118 ± 0.0026
Breasts	N/A	0.108 ± 0.0020
Esophagus	0.139 ± 0.0017	0.155 ± 0.0021
Eyes	0.094 ± 0.0008	0.118 ± 0.0026
Gallbladder Wall	0.202 ± 0.0051	0.207 ± 0.0006
Left colon	0.143 ± 0.0015	0.176 ± 0.0040
Small Intestine	0.143 ± 0.0012	0.162 ± 0.0031
Stomach Wall	0.145 ± 0.0015	0.178 ± 0.0030
Right colon	0.150 ± 0.0010	0.179 ± 0.0031
Rectum	0.134 ± 0.0012	0.167 ± 0.0046
Heart Wall	0.150 ± 0.0015	0.180 ± 0.0040
Kidneys	0.171 ± 0.0038	0.210 ± 0.0056
Liver	0.275 ± 0.0131	0.331 ± 0.0135
Lungs	0.167 ± 0.0061	0.207 ± 0.0072
Ovaries	N/A	0.171 ± 0.0040
Pancreas	0.161 ± 0.0015	0.210 ± 0.0015
Prostate	0.131 ± 0.0012	N/A
Salivary Glands	0.113 ± 0.0012	0.124 ± 0.0036
Red Marrow	0.113 ± 0.0010	0.135 ± 0.0031
Osteogenic Cells	0.167 ± 0.0015	0.200 ± 0.0045
Spleen	0.211 ± 0.0083	0.260 ± 0.0110
Testes	0.101 ± 0.0012	N/A
Thymus	0.126 ± 0.0021	0.162 ± 0.0040
Thyroid	0.121 ± 0.0017	0.132 ± 0.0030
Urinary Bladder Wall	0.130 ± 0.0012	0.154 ± 0.0040
Uterus	N/A	0.168 ± 0.0040
Whole body	0.112 ± 0.0012	0.143 ± 0.0031
Effective dose, mSv/MBq	0.1294 ± 0.0012	0.1721 ± 0.0028

Efficacy of ^{225}Ac-DOTA-hTAB004

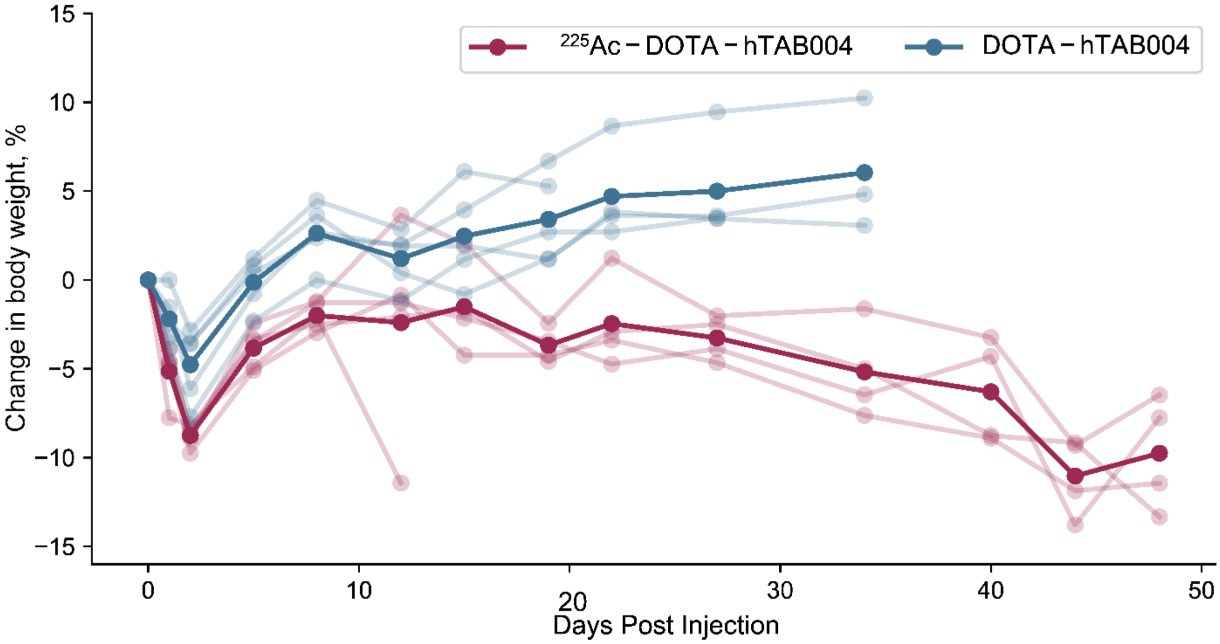

Figure S4: Percentage change in body weight from the day of dosing. Top: Percentage change in body weight from day of dosing (Darkened line: Group mean; fainter lines: Individual data). The ^{225}Ac-DOTA-hTAB004 group showed a decline in body weight from ~day 20 post dosing decreasing to 9.7% mean body weight decrease at day 48. In comparison the DOTA-hTAB004 group had an increase in body weight of 6% at study day 34.

Table S7: Statistical comparisons of the percentage change in tumor volume between day -2 and day 34.

**Study Day**	**Percentage change in Tumor volume, p values**		
	**<u>*^{225}Ac-DOTA-hTAB004 vs. DOTA-hTAB004*</u>**<br><br>*Inter-group comparisons Unpaired Student's T-Test*	*^{225}Ac-DOTA-hTAB004 group day -2 vs later study day*<br><br>*Intra-group comparisons Paired Student's T-Test.*	*DOTA-hTAB004 group day -2 vs later study day.*<br><br>*Intra-group comparisons Paired Student's T-Test*
day -2	**NS**	Not performed	Not performed
day 1	**0.032**	NS	0.025
day 5	**0.046**	NS	0.028
day 8	**0.029**	NS	0.028
day 12	**0.014**	NS	0.044
day 15	**0.003**	NS	0.028
day 19	**NS***	NS*	NS*
day 22	**<0.001**	NS	0.028
day 27	**<0.001**	NS	0.025
day 34	**<0.001**	0.013	0.028
day 37	**Not performed**	0.004	Not performed
day 40	**Not performed**	0.004	Not performed
day 44	**Not performed**	0.004	Not performed
day 48	**Not performed**	0.004	Not performed

* Large variation in tumor volumes at day 19 resulting in no significant differences at that timepoint.

Bibliography

1. OLINDA/EXM 2.0: The new generation dosimetry modeling code [Internet]. [cited 7 July 2019]. Available at: http://jnm.snmjournals.org/content/53/supplement_1/585

2. Kirschner AS, Ice RD, Beierwaltes WH. Radiation Dosimetry of 131I-19-Iodocholesterol: The Pitfalls of Using Tissue Concentration Data—Reply. J Nucl Med. 1975; 16: 248–9.
